# Comparing Cerebellar tDCS and Cerebellar tACS in Neurodegenerative Ataxias Using Wearable Sensors: A Randomized, Double-Blind, Sham-Controlled, Triple-Crossover Trial

**DOI:** 10.1007/s12311-023-01578-6

**Published:** 2023-06-22

**Authors:** Ilenia Libri, Valentina Cantoni, Alberto Benussi, Jasmine Rivolta, Camilla Ferrari, Roberto Fancellu, Matthis Synofzik, Antonella Alberici, Alessandro Padovani, Barbara Borroni

**Affiliations:** 1 Neurology Unit, Department of Clinical and Experimental Sciences, University of Brescia, Brescia, Italy; 2 Neurology Unit, Department of Neurological and Vision Sciences, ASST Spedali Civili, Brescia, Italy; 3 Department of Neuroscience, Psychology, Drug Research and Child Health (NEUROFARBA), University of Florence, Florence, Italy; 4 UO Neurologia, IRCCS Ospedale Policlinico San Martino, 16132 Genoa, Italy; 5 Department of Neurodegeneration, Hertie Institute for Clinical Brain Research and Centre of Neurology, Tübingen, Germany; 6 German Research Center for Neurodegenerative Diseases (DZNE), Tübingen, Germany

**Keywords:** cerebellar Ataxia, Transcranial direct current Stimulation, Transcranial alternating current stimulation, Wearable sensors

## Abstract

Cerebellar transcranial direct current stimulation (tDCS) represents a promising therapeutic approach for both motor and cognitive symptoms in neurodegenerative ataxias. Recently, transcranial alternating current stimulation (tACS) was also demonstrated to modulate cerebellar excitability by neuronal entrainment. To compare the effectiveness of cerebellar tDCS vs. cerebellar tACS in patients with neurodegenerative ataxia, we performed a double-blind, randomized, sham controlled, triple cross-over trial with cerebellar tDCS, cerebellar tACS or sham stimulation in twenty-six participants with neurodegenerative ataxia. Before entering the study, each participant underwent motor assessment with wearable sensors considering gait cadence (steps/minute), turn velocity (degrees/second) and turn duration (seconds), and a clinical evaluation with the scale for the Assessment and Rating of Ataxia (SARA) and the International Cooperative Ataxia Rating Scale (ICARS). After each intervention, participants underwent the same clinical assessment along with cerebellar inhibition (CBI) measurement, a marker of cerebellar activity. The gait cadence, turn velocity, SARA, and ICARS significantly improved after both tDCS and tACS, compared to sham stimulation (all *p*<0.010). Comparable effects were observed for CBI (*p*<0.001). Overall, tDCS significantly outperformed tACS on clinical scales and CBI (*p*<0.01). A significant correlation between changes of wearable sensors parameters from baseline and changes of clinical scales and CBI scores was detected. Cerebellar tDCS and cerebellar tACS are effective in ameliorating symptoms of neurodegenerative ataxias, with the former being more beneficial than the latter. Wearable sensors may serve as rater-unbiased outcome measures in future clinical trials. ClinicalTrial.gov Identifier: NCT05621200.

**Supplementary Information:**

The online version contains supplementary material available at 10.1007/s12311-023-01578-6.

## Introduction

Clinical treatments for neurodegenerative ataxias are hampered by the extreme heterogeneity of the underlying pathogenetic mechanisms and disease presentations. In this view, non-invasive cerebellar stimulation has been demonstrated to modulate cerebellar excitability and improve motor symptoms in patients with several different neurodegenerative cerebellar ataxias [1–3]. In particular, cerebellar transcranial direct current stimulation (tDCS) has been demonstrated to improve both motor outcomes at long-term and cognitive performances in patients with ataxia [4]. The treatment with cerebellar or cerebello-spinal tDCS has now proven to be safe, non-invasive, and have significant impact on motricity, cognition, and quality of life [5–7]. Moreover, cerebellar tDCS is able to restore cerebellar inhibition (CBI), a marker of cerebellar activity, measured by transcranial magnetic stimulation (TMS) [8, 9].

Important concepts are however emerging in the scientific field of cerebellar physiology and non-invasive brain stimulation techniques. Cerebellar ataxias are characterized by a predominant and early loss of Purkinje cells, which have been shown to oscillate in the gamma frequency band (30–80 Hz) [10]. In general, the gamma rhythm appears to function as a temporal code in cortex, facilitating the dynamic formation of neuronal assemblies by permitting synchronous firing among multiple, spatially separate subpopulations of neurons [11]. In this view, transcranial alternating current stimulation (tACS) is a novel non-invasive brain stimulation technique that has shown to enhance cortical oscillations by entraining brain rhythms to desired frequencies underneath the stimulated site. Animal models have confirmed that tACS may indeed entrain neuronal populations at specific frequencies [12]. Modulation of cortical cerebellar oscillations has been successfully applied in healthy subjects and has shown to induce cerebellar plasticity [13, 14]. Recent studies from our group have shown that tACS is safe and may be effective also in other neurodegenerative disorders [15, 16].

No study has compared the efficacy of cerebellar tDCS and cerebellar tACS yet, to identify the best stimulation paradigm among non-invasive stimulation techniques in neurodegenerative ataxias.

In this context, wearable technology is an increasingly popular method to monitor ataxic gait, with high sensitivity to small differences in disease severity, and may represent a promising and objective outcome markers in intervention trials [17, 18].

The above observations prompted the present study, aimed at assessing which technique is superior in improving motor outcomes. To achieve this, we carried out a randomized, double-blind, placebo-controlled, triple cross-over pilot study with cerebellar tDCS, cerebellar gamma tACS and sham stimulation, comparing clinical and digital-motor outcomes using highly sensitive state-of-the-art sensor-based motor and gait assessments.

## Methods

### Standard Protocol Approvals, Registrations, and Patient Consents

Full written informed consent was obtained from all participants according to the Declaration of Helsinki. The study protocol was approved by the local ethics committee (Brescia Hospital), #NP4514. The trial was registered at ClinicalTrials.gov (NCT05621200).

### Participants

Twenty-six participants with neurodegenerative ataxia, of whom eleven with a genetic form of spinocerebellar ataxia (five SCA1, five SCA2, one SCA38) [19], two with multiple system atrophy with cerebellar phenotype (MSA-C) [20], one with ataxia with oculomotor apraxia type 2 (AOA2) [21], and twelve with a sporadic adult-onset ataxia (SAOA) [22], were recruited from the Centre for Neurodegenerative Disorders, Neurology Unit, University of Brescia, Italy, and included in the study. Each patient fulfilled current clinical and/or genetic criteria for each specific diagnosis.

The diagnostic evaluation included past medical history, a neurological examination, standardized assessment of cerebellar functions, motor analysis using wearable sensors, and CBI assessment with TMS.

History of epilepsy, the presence of metal implants or electrical stimulators (i.e., pacemaker), pregnancy, orthopedic or visual dysfunction, and cognitive decline were considered exclusion criteria. Moreover, all patients had to be ambulatory and not yet in advanced disease stage (SARA <15 points).

### Study Design

We performed a randomized, double-blind, sham-controlled, triple cross-over study.

Participants were randomized into three groups in a 1:1:1 ratio (see Supplementary Figure 1). Group 1 received a single session of anodal cerebellar tDCS (real tDCS), group 2 a single session of gamma-tACS over cerebellum (real tACS), and group 3 placebo (sham) stimulation (T0). After 1 (T1) and 2 weeks (T2), stimulation was rotated, so that each patient underwent all of the three stimulations in a randomized order. According to literature data [7, 23], stimulation duration was different between protocols, with real tACS lasting 60 min and real tDCS lasting 20 min; however, to make stimulations indistinguishable by the participants, each stimulation (real tACS, real tDCS, and sham stimulation) was kept in place for 60-min overall.

At baseline (pre-stimulation), each patient underwent a neurological evaluation according to a standardized protocol including gait and balance analysis using wearable sensors (see gait and balance analysis as reported below), and a clinical evaluation with the scale for the assessment and rating of ataxia (SARA) [24] and the international cooperative ataxia rating scale (ICARS) [25] (see below, clinical assessment).

After each intervention (T0), and at 1- (T1) and 2-week (T2) follow-up, the same assessments were carried out, in addition to CBI evaluation with TMS (see CBI assessment).

The participants and the examiner performing clinical and wearable sensors evaluation (I.L.), tDCS/tACS, and TMS protocols (V.C.) were blinded to the type of stimulation. The device was previously set to real or sham stimulation by a different researcher. The clinical rating was video-recorded and analyzed randomly retrospectively by a blinded neurologist (A.A.). Inter-class coefficient between the two examiners (I.L. and A.A.) was 0.89 and 0.88 for SARA and ICARS scores, respectively. AA scoring was taken for final analyses.

B.B. was responsible for random allocation sequences, enrolment of participants, and assigned participants to specific interventions. A computer-assisted randomization was used to randomize subjects into groups.

### Outcome Measures

The primary endpoint was to establish the effectiveness of cerebellar tDCS and cerebellar gamma tACS, and which non-invasive brain stimulation technique may be most effective in improving motor symptoms in patients with cerebellar ataxia using the SARA scale. The secondary endpoints were defined as changes on the ICARS, state-of-the-art wearable sensors, and neurophysiological parameters of CBI, evaluated with TMS.

### Gait and Balance Analysis

At each time point, participants performed a standardized battery to assess walking and balance. Three Opal inertial sensors (APDM, Inc., Portland, WA) were attached on both feet and posterior trunk at the level of L5 with elastic Velcro bands. Inertial sensor data were collected and wirelessly streamed to a laptop for automatic generation of gait and balance metrics by Mobility Lab software (APDM, Inc.) [17].

For the purpose of the present study, and based on previous studies on gait measures in degenerative ataxia [17, 26–29], we used the following parameters: (a) mean left and right lower limbs cadence (gait cadence, steps/minute), i.e., number of steps walking for two minutes in a quiet indoor floor, and supervised by a study assessor; patients were instructed to walk normally at a self-selected speed in a flat corridor, 10 m long of each gait bout, and 3 m broad, with no other people present; (b) turn velocity (degrees/second), namely the speed for a 360° turn; and (c) turn duration (seconds), namely the time needed for a 360° turn; patients stood with their toes aligned with the tape on the floor to mark the start/stop position; the patient was asked to complete a full turn, with the subject’s shoulders were back in the start position. For each parameter, we considered the change after each intervention as compared to baseline.

### Clinical Assessment

A standardized assessment of cerebellar functions was performed by means of SARA [24] and ICARS [25]. SARA consists of eight items, including gait, stance, sitting, speech disturbance, finger chase, nose-finger test, fast alternating hand movements, and heel-shin slide. ICARS is a semiquantitative 100-point scale consisting of 19 items, divided into four weighted sub-scores, namely posture and gait disturbances, limb kinetic function, speech disorder, and oculomotor deficits. For each score, the higher the score level, the worse is the patient’s performance.

### Cerebellar Inhibition

Two monophasic Magstim TMS stimulators connected with two 70-mm figure-of-eight coils (Magstim Company, Oxford, UK) were used to evaluate CBI, as previously published [30].

Surface Ag/AgCl electrodes positioned in a belly-tendon montage on the right first interosseous muscles (FDI) were used to record motor evoked potentials (MEPs), using a Biopac MP-150 electromyograph (BIOPAC Systems Inc., Santa Barbara, CA, USA). Responses were amplified and filtered at 20 Hz and 2 kHz with a sampling rate of 5 kHz.

The stimulation coil was positioned with the handle directed 45° laterally and posteriorly to the sagittal plane, over the region corresponding to the primary motor cortex (hand area), contralateral to the target FDI. The “hot spot” was defined as the point in which magnetic stimulation resulted in the maximum motor evoked potential (MEP) amplitude with the minimum stimulator intensity, which was marked with a felt tip pen on the scalp to ensure constant placement of the coil throughout the experiment [31].

RMT was defined as the minimal stimulus intensity needed to produce MEPs with an amplitude of at least 50 μV in 5 out of 10 consecutive trials during complete muscle relaxation, which was controlled by visually checking the absence of EMG activity at high-gain amplification [32].

CBI was assessed using previously described techniques [33–35]. Briefly, the second coil was used to deliver the conditioning stimuli (CS) which was placed over the contralateral cerebellar hemisphere (1 cm inferior and 3 cm right to the inion), a site corresponding to the posterior and superior lobules of the lateral cerebellum [36]. For cerebellar stimulation, the handle was positioned upward with the coil placed tangentially to the skull. The cerebellar CS intensities were set at 110% RMT obtained in the contralateral motor cortex [33]. CS preceded the target stimuli (TS) by different interstimulus intervals (ISIs) ranging from 3 to 10 ms (3, 5, 10 ms). There were four conditions, corresponding to the three different ISI and the TS alone. Ten responses were collected for each different ISI and fifteen for the TS alone in a pseudorandomized sequence. The amplitude of the conditioning MEPs was expressed as a ratio of the mean unconditioned response. The inter trial interval was set at 5 s (±10%).

### Non-invasive Brain Stimulation Interventions

#### Transcranial Direct Current Stimulation

Transcranial direct current stimulation (tDCS) was delivered by a battery-driven constant current stimulator (Brainstim, EMS, Italy) through a pair of saline-soaked (0.9% NaCl) surface sponge electrodes (7×5 cm^2^, current density 0.057 mA/cm^2^ for the anodal cerebellar electrode: 8×6 cm^2^, current density 0.042 mA/cm^2^ for the cathodal electrode). The anode was placed on the scalp over the cerebellum area (2 cm under the inion) and the cathode over the right deltoid muscle. We opted for this specific montage as opposed to other montages (e.g., cerebello-spinal montage) for the following reasons: we aimed to reduce potential confounding factors by focusing on a single target area for stimulation, thus controlling for extra variations that multiple stimulation sites might introduce. This approach enables us to attribute any detected changes in cerebellar activity and associated motor outcomes more directly to the tDCS and tACS interventions. Furthermore, the impact of tACS on the spinal cord remains poorly understood. A particular concern was that gamma stimulation via tACS over the spinal cord could possibly worsen motor function.

The electrodes were secured using elastic gauzes and an electroconductive gel was applied to electrodes to reduce contact impedance (<5 kΩ for all sessions). To ensure blindness, the session lasted 60 min, but the real anodal stimulation, a constant current of 2 mA, was applied only for the last 20 min, as suggested by recently published consensus recommendations [7, 23].

#### Gamma Transcranial Alternating Current Stimulation (gamma-tACS)

A single session of tACS was delivered by the same device as tDCS with identical electrode placement. An alternating sinusoidal current of 1.5 mA peak-to-baseline (3.0 mA peak-to-peak, current density: 0.031 mA/cm2) at a frequency of 40 Hz was applied for 60 min.

#### Placebo Stimulation (Sham)

For the sham condition, the device and electrode placement were the same, but the electric current was ramped down 60 s after the beginning of the stimulation to make this condition indistinguishable from the experimental stimulation. To detect differences in the perception of the stimulation, we asked the patients whether they thought they were receiving real or sham stimulation at the end of each session; no significant difference in sensations was reported.

### Statistical Analyses

We used a power analysis to determine the necessary sample size of the primary outcome, based on previously published work on tDCS in ataxia [1–4, 37]; considering power (1-beta = 0.80) and alpha = 0.05, we calculated that twenty-six patients would be needed, correcting also for possible dropouts, with an estimated drop-out rate of 10% observed in similar studies in the same setting [2, 3, 8].

Data are expressed as mean and standard deviation, unless otherwise stated. To assess the effect of tDCS or tACS treatment on clinical and instrumental scores, we used a one-way ANOVA. Only when a significant main effect was reached, post hoc tests with Bonferroni correction for multiple comparisons were conducted to analyse group-differences. Pearson’s correlation analysis was carried out to correlate clinical and neurophysiological scores. Statistical analyses were performed using SPSS version 21 (SPSS, Inc., Chicago, IL, USA).

## Results

### Participants

Demographic and clinical features of included patients are reported in Table 1. Twenty-six participants (mean age: 52.5±13.9 years, female sex: 57%, disease duration: 6.9±5.5 years) were enrolled. Every participant completed all clinical evaluations, except one patient who did not complete T2 for lumbar fracture.

**Table 1:** Demographic and clinical features of included patients

Patient	Age, y	Age at onset, y	Disease duration, y	Sex	Diagnosis	SARA	ICARS
1	42	35	7	M	SCA1	6	15
2	50	40	10	F	SCA1	3	7
3	28	7	21	F	AOA2	21	52
4	44	36	8	M	SCA1	11.5	29
5	49	43	6	F	SAOA	9	23
6	34	32	2	F	SAOA	5	9
7	42	41	1	M	SAOA	4	13
8	51	47	4	F	SAOA	8	18
9	44	39	5	F	SCA2	12	28
10	55	49	6	F	SAOA	3	11
11	23	20	3	F	SCA2	14	38
12	70	68	2	M	MSA-C	12	23
13	59	57	2	F	SAOA	13.5	26
14	68	45	23	F	SCA38	16	38
15	56	54	2	F	SCA2	4	13
16	73	61	12	F	MSA-C	10	21
17	59	50	9	M	SCA2	7	16
18	59	57	2	M	SAOA	12	27
19	48	45	3	M	SAOA	13	38
20	42	35	7	M	SCA1	8	19
21	58	46	12	M	SCA2	12	28
22	80	71	9	M	SAOA	5	12
23	68	64	4	F	SAOA	4.5	8
24	65	61	4	F	SAOA	3	6
25	39	34	5	M	SCA1	9	17
26	59	50	9	F	SAOA	10	21

*SCA* spinocerebellar ataxia, *AOA2* ataxia with oculomotor apraxia type 2, *MSA-C* cerebellar variant of multiple system atrophy, *SAOA* sporadic adult-onset ataxia, *SARA* Scale for the Assessment and Rating of Ataxia, *ICARS* International Cooperative Ataxia Rating Scale, *M* male, *F* female, *y* years

### Motor Assessment

**Fig. 1:**
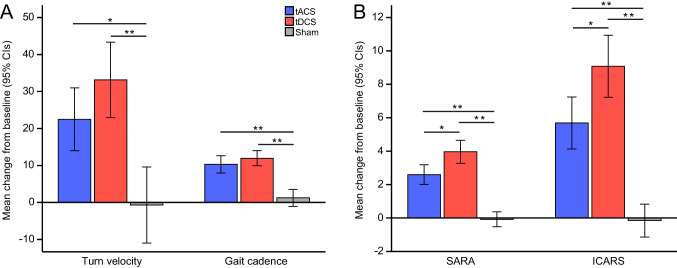
Changes in **A** wearable sensors parameters and **B** SARA and ICARS scores compared to baseline after tACS, tDCS, or sham stimulation. LL = lower limbs; tACS = transcranial alternating current stimulation; tDCS = transcranial direct current stimulation; SARA = Scale for the Rating of Ataxia; ICARS = International Cooperative Ataxia Rating Scale. Higher values mean better performances after stimulation. *p<0.01; **p<0.001; error bars represent 95% confidence intervals

We considered changes of wearable sensors parameters and clinical scales after each intervention compared to baseline scores (see Table 2 and Fig. 1; for individual values see Supplementary Fig. 2), positive higher mean values indicating better performances after stimulation.

**Table 2:** Changes of wearable sensors scores and clinical scales after non-invasive brain stimulation interventions

Variable	Baseline score (95%CI)	ΔtACS (95%CI)	ΔtDCS (95%CI)	Δsham (95%CI)	p*
*Clinical scales*					
SARA	9.1 (7.2–10.9)	2.6 (2.0–3.2)	4.0 (3.3–4.6)	−0.1 (−0.5–0.3)	<0.001
ICARS	21.4 (16.8–25.9)	5.5 (4.1–7.2)	9.1 (7.2–10.9)	−0.1 (−1.1–0.8)	<0.001
*Wearable sensors*					
LL cadence, steps/min	109.5 (103.3–115.7)	10.3 (8.0–12.6)	12.0 (9.9–14.0)	1.2 (−1.1–3.5)	<0.001
Turns velocity, degrees/sec	148.8 (126.6–170.9)	22.5 (13.9–31.0)	33.1 (22.9–43.3)	−0.7 (−10.1–9.6)	<0.001
Turns duration, sec	4.3 (3.7–4.9)	0.5 (0.1–1.0)	1.1 (0.6–1.7)	0.2 (−0.4–0.7)	0.03

*tACS* transcranial alternating current stimulation, *tDCS* transcranial direct current stimulation, *LL cadence* mean left and right lower limbs cadence, *SARA* Scale for the Assessment and Rating of Ataxia, *ICARS* International Cooperative Ataxia Rating Scale, *sec* seconds, *min* minutes

Δ = [baseline score – post treatment score]; results are expressed as mean (95% confidence intervals)

*p-*values: Δ baseline comparisons; *p*<0.01 was considered significant, after corrections for multiple comparisons

#### Wearable Sensors

Gait cadence (steps/minute) was significantly different after tDCS compared to sham stimulation, with a mean difference of 12.0 (95%CI, 9.9 to 14.0, *p*<0.001); gait cadence was also significantly different after tACS compared to sham stimulation, with a mean difference of 10.3 (95%CI, 8.0 to 12.6, *p*<0.001); tDCS and tACS were not significantly different from each other (*p*=0.828).

Comparably, turn velocity (degrees/second) was significantly different after tDCS compared to sham stimulation, with a mean difference of 33.1 (95%CI, 22.9 to 43.3, *p*<0.001), and it was also significantly different after tACS with a mean difference of 22.5 (95%CI, 13.9 to 31.0, *p*=0.003); tDCS and tACS were not significantly different from each other (*p*=0.338).

When turn duration (seconds) was considered, comparisons did not survive to multiple corrections.

#### Clinical Scales

Clinical ataxia scores were significantly different after tDCS compared to sham stimulation, with a mean difference of 4.0 (95%CI, 3.3 to 4.6, *p*<0.001) in the SARA scale and 9.1 (95%CI, 7.2 to 10.9, *p*<0.001) in the ICARS scale; clinical ataxia scores were also significantly different after tACS compared to sham stimulation, with a mean difference of 2.6 (95%CI, 2.0 to 3.2, *p*<0.001) in the SARA scale and 5.5 (95%CI, 4.1 to 7.2, *p*<0.001) in the ICARS scale. Furthermore, we observed a significant increase in ataxia scores changes after tDCS compared to tACS, for both SARA (*p*=0.003) and ICARS (*p*=0.005).

### Cerebellar Inhibition

**Fig. 2:**
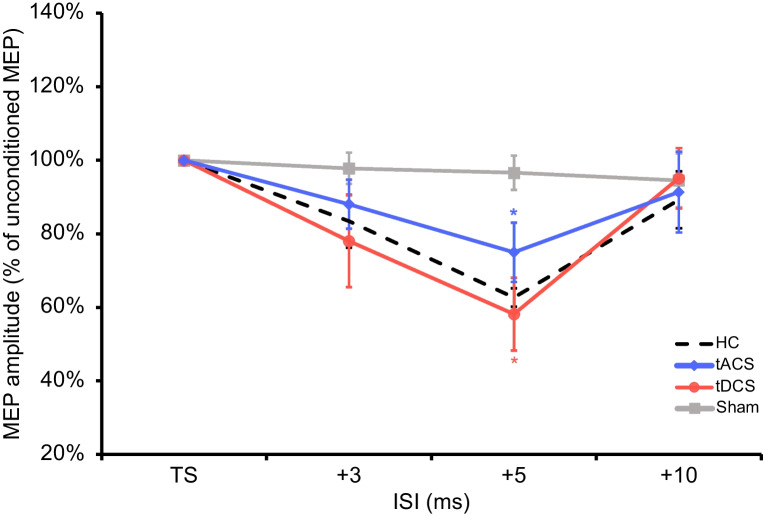
Cerebellar inhibition (CBI) scores after tACS, tDCS, or sham stimulation. HC = 10 age- and gender-matched healthy controls, as reference group; tACS = transcranial alternating current stimulation; tDCS = transcranial direct current stimulation. *p<0.001, tACS vs. sham, tDCS vs. sham, and tDCS vs. tACS; error bars represent 95% confidence intervals

Cerebellar inhibition (CBI) was available for 21 out of 26 patients. As reported in Fig. 2, lower peak CBI (5 ms) values (e.g., increased inhibition, closer to healthy controls’ values) were found after tDCS (0.58±0.1) and tACS (0.75±0.2) stimulations compared to sham stimulation (0.97±0.1, both *p*<0.001). A significant difference was also reported between tDCS and tACS stimulations (*p*<0.001).

### Correlation Between Wearable Sensors Scores and Clinical Or Neurophysiological Data

Changes of gait cadence from baseline (steps/minute) significantly correlated with changes of SARA (*r*=0.44, *p*<0.001) and ICARS (*r*=0.42, *p*<0.001), the greater the improvement of gait cadence, the greater the amelioration of ataxia scales scores.

Comparable results were obtained when changes of turn velocity (degrees/second) were correlated to changes of SARA (*r*=0.40, *p*=0.001) and ICARS (*r*=0.37, *p*=0.001).

**Fig. 3:**
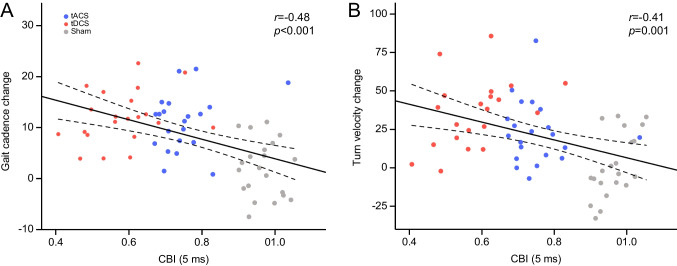
Correlation analyses between sensor-based measures of **A** gait cadence change and **B** turn velocity change and cerebellar inhibition (CBI) scores after intervention. tACS = transcranial alternating current stimulation; tDCS = transcranial direct current stimulation; dashed lines represent 95% confidence intervals

Changes of gait cadence and changes of turn velocity significantly correlated with CBI scores after treatment (*r*=−0.48, *p*<0.001 and *r*=−0.41, *p*=0.001, respectively), the greater the improvement of wearable sensors scores the lower the CBI score after treatment intervention (e.g., closer to healthy controls’ values) (see Fig. 3).

## Discussion

In this study, we reported the effectiveness of both cerebellar tDCS and cerebellar tACS as intervention approaches in neurodegenerative ataxias, with the former outperforming the latter. We tested our hypothesis considering sensor-based assessments, clinical scales, and measures of cerebellar connectivity.

Non-invasive brain stimulation techniques have gained much attention in the recent years, and an increasing body of literature has demonstrated their efficacy in neurodegenerative disorders [38–43]. However, different diseases are likely to benefit from tailored approaches, and the best of these has yet to be established.

In neurodegenerative ataxias the efficacy of cerebellar tDCS, which acts on synaptic plasticity, has consistently been reported [2, 4, 8, 9]. Conversely, tACS may be considered as a novel therapeutic intervention able to entrain specific brain rhythms, as Purkinje cells have been shown to oscillate in the gamma frequency band (30–80 Hz) [10].

In this view, sensor-based assessments have the potential to complement existing clinical assessment techniques, offering advantages in terms of objectivity and eliminating inter-rater variabilities in assessing ataxia [44, 45]. To this, turning movements are particularly challenging for dynamic balance control and walking behavior, such as lower limbs cadence, should be able to capture clinically important differences and relevant changes in patient-centered outcome measures [18]. By unbiased parameters derived from wearable sensors, we confirmed the efficacy of cerebellar tDCS in neurodegenerative ataxias and we reported for the first time the effects of cerebellar tACS. We did not find significant differences between the two approaches, even though we may suggest a trend supporting cerebellar tDCS. The preferential choice of cerebellar tDCS was also supported by significant differences in clinical ataxia scales, namely SARA and ICARS, and in cerebellar-brain connectivity measures. Once again, we demonstrated that both approaches are highly effective in ameliorating ataxia symptoms but tDCS performed better than tACS.

The consistency of the present results was further corroborated by significant correlations between wearable sensors parameters, clinical scales, and cerebellar-brain connectivity measure.

We acknowledge that this study entails some limits. First, these findings are limited by our study cohort not being sufficiently powered for stratification according to specific ataxia genotypes. Second, we considered only a few parameters derived by wearable technology and more detailed and ataxia-specific measures might be assessed in the next future. Lastly, we did not evaluate if different stimulation protocols may have different long-term effects by means of repeated stimulations.

However, our study allowed us to answer several clinical questions; it strongly supports the usefulness of non-invasiveness brain stimulation techniques in neurodegenerative ataxias; it compared two different approaches suggesting cerebellar tDCS as the best therapeutic intervention candidate in these disorders; and it extended the current literature claiming for the usefulness of digital-motor biomarkers as outcome measures for future treatments trials.

### **Supplementary Information**

ESM 1

## Data Availability

All data, including outcome measure results, study protocol and statistical analysis plan, will be shared through ClinicalTrials.gov via public access (https://clinicaltrials.gov/ct2/show/ NCT05621200).
