# Supplementary material for: Comparing Cerebellar tDCS and Cerebellar tACS in Neurodegenerative Ataxias Using Wearable Sensors: A Randomized, Double-Blind, Sham-Controlled, Triple-Crossover Trial
Source: Cerebellum. 2023 Jun 22;23(2):570–8. doi: 10.1007/s12311-023-01578-6 (PMC10951038; doi:10.1007/s12311-023-01578-6)
Supplement: Supplementary file 1 — ESM 1 [file 12311_2023_1578_MOESM1_ESM.pdf]

Supplementary Figure 1. Flow chart of the study.

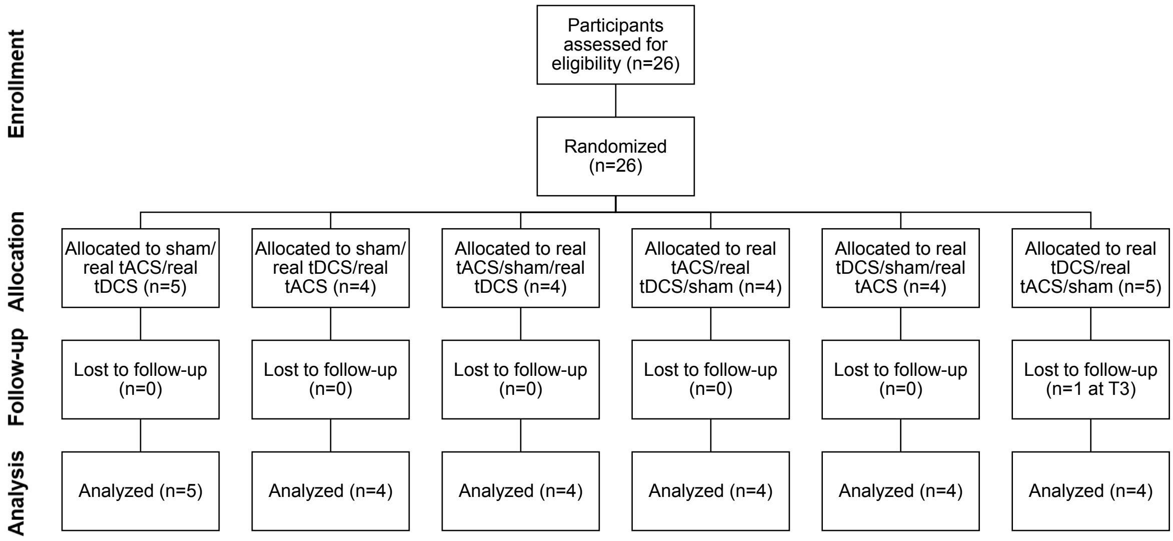

Legend: tACS = transcranial alternating current stimulation; tDCS = transcranial direct current stimulation.

Supplementary Figure 2. Changes in wearable sensors parameters, SARA and ICARS scores compared to baseline after tACS, tDCS or sham stimulation. Individual values are represented.

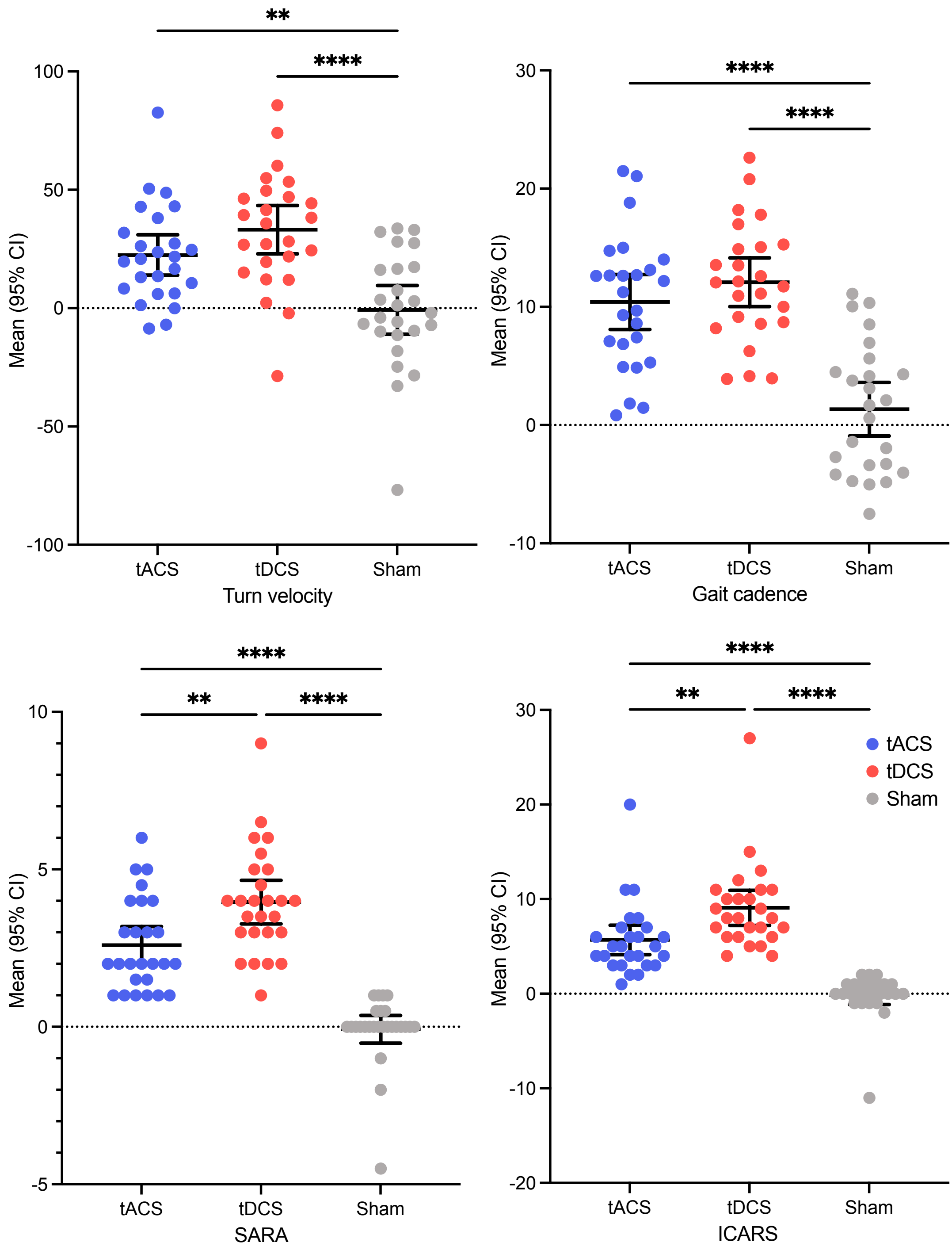

Legend: tACS = transcranial alternating current stimulation; tDCS = transcranial direct current stimulation; SARA = scale for the rating of ataxia; ICARS = international cooperative ataxia rating scale.

***p<0.01; ***p<0.001; ****p<0.0001; Long bars represent mean values while error bars represent 95% confidence intervals.
